# SI ATRP for the Surface Modifications of Optically Transparent Paper Films Made by TEMPO-Oxidized Cellulose Nanofibers

**DOI:** 10.3390/polym14050946

**Published:** 2022-02-26

**Authors:** Jem-Kun Chen, Hsiang-Ya Huang, Cheng-Wei Tu, Li-Ting Lee, Tongsai Jamnongkan, Chih-Feng Huang

**Affiliations:** 1 Department of Materials Science and Engineering, National Taiwan University of Science and Technology, Taipei 10607, Taiwan; jkchen@mail.ntust.edu.tw; 2 Department of Chemical Engineering, i-Center for Advanced Science and Technology (iCAST), National Chung Hsing University, Taichung 40227, Taiwan; sylvia19971118@gmail.com; 3 Industrial Technology Research Institute, Chutung, Hsinchu 31057, Taiwan; cwtu670302@gmail.com; 4 Department of Materials Science and Engineering, Feng Chia University, Taichung 40724, Taiwan; ltlee@fcu.edu.tw; 5 Department of Fundamental Science and Physical Education, Faculty of Science at Sriracha, Kasetsart University, Bangkok 10900, Thailand

**Keywords:** TOCN, SI ATRP, optically transparent paper film, polystyrene

## Abstract

Applications of cellulose nanofibers currently match the demands of biodegradable and renewable constituent biocomposites. In this study, we studied the process of preparing TEMPO-oxidized cellulose nanofibers (TOCNs). These nano-sized cellulose fibers (ca. 11 nm) can be fabricated to high transmittance and optically transparent paper (OP) films. Then the OP films can be facilely immobilized initiating sites for the subsequent surface-initiated atom transfer radical polymerization (SI ATRP). We investigated SI ATRP with styrene (St) kinetics and monitored chemical structure changes of the OP surfaces. The obtained OP-*g*-PSt significantly led to enhance thermal stability and alter the OP surface with hydrophobic compared to that of pristine OP film. Characterization was studied by Fourier transform infrared spectroscopy (FT-IR), powder X-ray diffraction (XRD), X-ray photoelectron spectroscopy (XPS), scanning electron microscopy (SEM), UV–Vis spectroscopy, thermogravimetric analyzer (TGA), and water contact angle (WCA) measurements.

## 1. Introduction

Cellulose is the most abundant natural material as well as an important renewable resource. Cellulose exists widely in plants, generated by bacteria and some animals from the polymerizations of glucose units. Most importantly, the cellulose in plants is generated via the photosynthesis process that is nowadays a substantial factor for the carbon capturing trend. The utilization of cellulose can not only suppress the greenhouse effect but also substitute for the oil-based products/feedstock. After photosynthesis or animal metabolism procedures, the generated celluloses are composed of glucose units with β-1,4-glycosidic linkages [1,2,3]. In the presence of dense hydrogen bonding, this analog of β-1,4-glycoside linked polysaccharides generate a high crystallinity microstructure and forms micrometer sized bundles. In recent decades, cellulose nanofibers have been further obtained and applied to nanofillers or nanofabrication for the explorations of emerging materials. According to the preparing methods, four common analogs of cellulose nanofibers have been classified: (i) 2,2,6,6-tetramethylpiperidine-1-oxyl (TEMPO)–oxidized cellulose nanofibrils (TOCNs) [1,4], (ii) cellulose nanowhiskers/cellulose nanocrystals (CNW/CNC) [5,6], (iii) bacterial nanocelluloses (BNC) [7], and (iv) mechanically fibrillated cellulose nanofibrils (CNF) [8]. Similar to the micrometer-sized natural cellulose fibers, these cellulose nanofibers remain high crystallinity. However, they possess significantly enhanced physical properties, such as widths in nanometers, high aspect ratios (i.e., 10–1000 for CNF; 50–100 for CNC; 100–500 for BNC and TOCN), high elastic moduli (i.e., 100–150 GPa), high tensile strengths (i.e., 7–10 GPa), large surface areas (i.e., 200–350 m^2^/g), and liquid crystal phases (in CNW analog).

One emerging application of cellulose nanofibers is to serve as a nanofiller for the preparations of polymer nanocomposites. For example, Isogai et al. prepared poly(vinyl alcohol) (PVA) and TOCN hybrid fibers and improved the maximum elongation up to 20 times compared to that of conventional neat PVA fibers [9]. From the same group, nanocomposite films containing TOCN and polystyrene (PSt) [10] and poly(l-lactide) (PLLA) [11], respectively, demonstrated significant improvements on tensile modulus/strength and thermal dimension stability compared to the pristine polymer films. In the case of PLLA/TOCN system, notably, one importance is that TOCNs played a role in the improvement of coefficient of thermal expansion (CTE). Another important role of TOCNs is to increase effective nucleation rate of PLLA that is quite critical for the practical PLLA processing applications. Ougizawa et al. [12] and Huang et al. [13] reported poly(methyl methacrylate) (PMMA) and surface-modified TOCNs nanocomposites. The PMMA/mTOCN nanocomposites demonstrated controllable birefringence and improvements in thermal and mechanical properties and retained excellent transparency. Several other nanocomposites including CNF, CNW, and BNC also demonstrate their hybridization effectiveness [14,15,16,17,18,19,20]. Another advantage of cellulose nanofibers is that they are able to fabricate free-standing optically transparent paper (OP) films [7] that should match the next generation consumer electronics to replace/suppress current electronics of non-biodegradable, oil-based products that contain toxic materials. With high demands from consumers, the discarded or upgraded electronics might cause our environmental encumbrance. Thus, electronic devices comprising less amounts of toxic contaminants and biodegradable and renewable constituents are correspondingly demanded. One breakthrough example was reported by Jung et al. [21]. They fabricated high-performance flexible microwave and digital electronics from OP films. The resulted green devices achieved nearly non-toxic, biodegradable, and flexible features. Based on an eco-friendly viewpoint, they successfully demonstrated the integrations of electrical parts on the flexible OP films, and they had commensurate physical properties [22].

Regarding the abovementioned practical applications, facile modifications of the hydrophilic surfaces from cellulose nanofibers are the keys to attain good dispersion in hydrophobic polymers as a nanofiller and suppress water adsorption drawbacks as an OP film. In recent decades, powerful tools of surface-initiated (SI) controlled/living radical polymerizations (LRPs) provide facile approaches for the functionalization of any organic or inorganic surfaces from macroscopic to microscopic [23,24,25]. Beneficial because of their robust features, one can conduct SI LRPs with a variety of monomers and tolerance of functionalities. More importantly, surface polymer chains with well-defined molecular weight (MW), low polydispersity (PDI), and various graft density can be attained. Thus, we can easily alter the surface properties, including hydrophilicity/hydrophobicity, anti-biofouling/bio-adhesions, etc. Numerous groups have demonstrated SI LRP studies, such as nitroxide-mediated radical polymerization (SI NMP) [26,27], reversible addition-fragmentation chain transfer (SI RAFT) polymerization [28], atom transfer radical polymerization (SI ATRP) [29,30,31,32], as well as other relevant methods [26,27,28]. A few studies further demonstrated SI LRPs from cellulose nanofibers. For instance, Zhang et al. conducted SI ATRP from CNW with styrene (St). They successfully obtained CNW-*g*-PSt nanohybrid materials and showed interesting thermotropic/lyotropic liquid crystal states [33] of the resulted nanomaterials. Thielemans et al. similarly had SI ATRP from CNW with St and efficiently applied the obtained nanomaterials to remove aromatic pollutants [34]. By performing SI ATRP from BNC with acrylic monomers, Barros-Thimmons et al. attained tunable mechanical, thermal, and hydrophobic properties of the BNC-based nanocomposites [35]. Utilizations of SI ATRP from OP films should, thus, enrich their practical applications, especially in the area of “green” electronics.

**Scheme 1:**
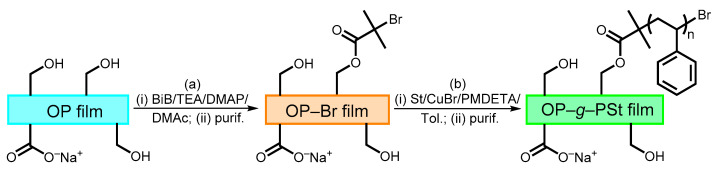
(a) Esterification of OP film with BiB in DMAc. (b) (SI) ATRP with St/HEBiB/CuBr/PMDETA(/OP-Br) in toluene (purif.: purification).

In this study, TOCN-based OP films are made first. As shown in Scheme 1a, immobilization of an ATRP initiating site can be attained via surface esterification to afford OP-Br films. As shown in Scheme 1b, subsequently, SI ATRP from the OP-Br films with St were conducted to alter the surface and physical properties of the films. This study was investigated by Fourier transform infrared spectroscopy (FT-IR), powder X-ray diffraction (XRD), X-ray photoelectron spectroscopy (XPS), scanning electron microscopy (SEM), UV–Vis spectroscopy, thermogravimetric analyzer (TGA), and water contact angle (WCA) measurements.

## 2. Materials and Methods

### 2.1. Materials

Details are depicted in the Electronic Supplementary Material (ESM).

### 2.2. Preparations of Optically Transparent Paper (OP) Films and Further Immobilizations of ATRP Initiating Sites (OP-Br)

The procedure for the preparations of 2,2,6,6-tetramethylpiperidine 1-oxyl (TEMPO) oxidized cellulose nanofibers (TOCNs) is depicted in the ESI. Degree of oxidation (DO) of the TOCNs was estimated by conductivity meter [36]. Preparations of OP films: The obtained TOCN sample was diluted by deionized water (DIW) to a concentration of 10 g/L and vigorous stirring was applied by a homogenizer for 2 min under nitrogen, following by further 5 min of high power ultra-sonication (200 W). The solution was filtrated by a nylon filter paper with a pore size of 20 μm. The filtrate was collected, poured into a Petri dish, and conducted air-dry for 2 days to afford OP films.

Immobilizations of ATRP initiating sites: An OP film (3 × 6 cm^2^), proper amounts of 4,4-dimethylaminopyridine (DMAP), and triethylamine (TEA) were placed in a reaction flask with anhydrous *N*,*N*-dimethylformamide (DMF, 50 mL). The reaction flask was placed in an ice bath and 2-bromoisobutyryl bromide (BiB) were added dropwise using an addition funnel. After the reaction was completed, saturated NH_4_OH_(aq)_ (50 mL) was added to neutralize the active species. The film was washed by CH_2_Cl_2_ and MeOH several times and dried in vacuum. Then the OP-Br film was obtained and stored in a desiccator.

### 2.3. SI ATRP from OP-Br with St in the Presence of Free Initator HEBiB

A free initiator of 2-hydroxyethyl 2-bromoisobutyrate (HEBiB) was prepared according to the literatures [37,38] in order to trace the molecular weight and PDI of the grafted polymer chains. HEBiB (22.5 μL, 0.159 mmol), *N*,*N*,*N*′,*N*″,*N*″-pentamethyldiethylenetriamine (PMDETA, 33.0 μL, 0.159 mmol) and OP-Br (3 × 2 cm^2^) were added to a Schlenk tubular flask with deoxygenated St (4.8 mL, 31.95 mmol), toluene (7.5 mL), and CuBr (St/HEBiB/CuBr/PMDETA = 200:1:1:1; [St]_0_ = 2.6 M) and sealed. A t_0_ sample was collected via syringe and then the flask was immersed in a shaking bath at 70 °C. During the (SI) ATRP, tracing samples were taken to analyze the reaction kinetics by gas chromatography (GC) and molecular weight (MW) characterization by gel permeation chromatography (GPC). The polymerization was terminated by placing the tubular reaction flask in an ice bath and exposing the contents to air. The modified OP film was washed with THF and MeOH to remove the free PSt homopolymer and residual catalyst and dried in vacuum. We then obtained OP-*g*-PSt hybrid films with high transparency.

### 2.4. Characterization

FT-IR spectra were collected by a PerkinElmer Spectrum One FT-IR spectrometer (64 scans at a resolution of 1 cm^−1^; PerkinElmer, MA, USA). Powder samples were mixed with KBr for bulk KBr disk preparations by a hydraulic press and the film samples were measured directly. X-ray diffraction (XRD) spectra were acquired in diffraction angles (2θ) from 10 to 30° using a Bruker AXS D8 Discover SSS high-resolution X-ray diffractometer (Bruker, MA, USA) and Ni-filtered Cu Kα radiation (*λ* = 0.1548 nm). Scanning electron microscope (SEM) of JEOL JSM 7401F FE-SEM (JEOL, Tokyo, Japan) was utilized to screen the microstructures with an accelerating voltage of 150 kV in order to collect the SEM images in a short period. X-ray photoelectron spectroscopy (XPS) was proceeded with a monochromated Al Kα X-ray source (*hv* = 1486.6 eV). UV–Vis measurements were measured using a Hitachi U-3900 spectrophotometer (HITACHI, Tokyo, Japan). Conversions of St monomer were traced by GC using solvent as the internal standard (Hewlett–Packard 5890 series II gas chromatograph equipped with an FID detector and employing a CNW CD-5 column; Hewlett Packard, Palo Alto, CA, USA). Acquired samples were analyzed by GPC to characterize MWs and polydispersities (PDIs) of PSt polymers (eluent: THF; flow rate: 1 mL/min at 40 °C; Waters 410 differential refractometer; PSS SDV columns of Linear S and 100 Å pore size; SHIMADZU, Kyoto, Japan). PSt standards were used for calibration. Thermal stability was measured by a TA Instruments Q50 (TA Instruments, New Castle, Germany) thermogravimetric analyzer featuring a platinum holder operated at a heating rate of 20 °C/min in the temperature range 25–800 °C under a N_2_ atmosphere. Water contact angles (WCAs) were examined using a KRÜSS G10 system (KRÜSS, Hamburg, Germany) with a water droplet of ca. 5 μm.

## 3. Results and Discussion

**Figure 1:**
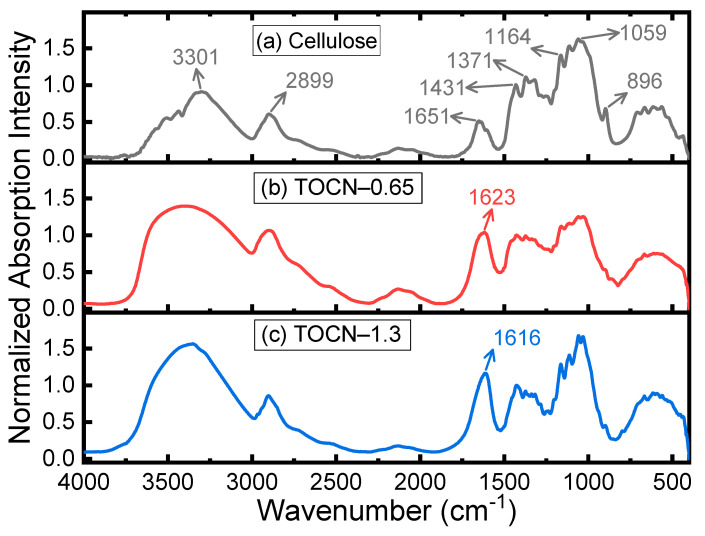
FT-IR spectra (4000–400 cm^−1^) of (**a**) cellulose pulp, (**b**) TOCN-0.65, and (**c**) TOCN-1.3.

The C6-OH moiety of glucose repeat units in pristine celluloses can be effectively oxidized and converted to COO^−^Na^+^ by TEMPO oxidation method, leading to the acquirement of TEMPO-oxidized nanocellulose (TOCN). During the oxidation process, the reduction agent of NaClO_(aq)_ plays as an important role in mediating the overall oxidation efficacy. With different amounts of reduction agent, herein, we examined and compared the influences with different initial concentrations of NaClO_(aq)_ (0.65 and 1.3 M) on TEMPO-oxidized celluloses in 4 h. The curve a in Figure 1 shows FT-IR spectrum of cellulose pulp. We observe cellulose feature peaks at 1651 cm^−1^ for the absorbed water vibrations and broad peaks of 3301 cm^−1^ for hydroxyl vibrations including intermolecular and intramolecular hydrogen bonding. The other features were commonly found in the structure of the cellulose D-glucose repeating unit, such as 896 cm^−1^ for C-O-C stretching, 1059 and 1164 cm^−1^ for C-C and C-O-C asymmetric stretching, 1371 cm^−1^ for CH bending, 1431 cm^−1^ for CH_2_ bending, and 2899 cm^−1^ for CH symmetrical stretching [39,40]. The curves b and c in Figure 1 display FT-IR spectra of TOCN-0.65 and TOCN-1.3 samples made by TEMPO oxidation method, revealing that characteristic absorption peaks have some distinguishable changes. It is mainly necessary to confirm whether the COO^−^Na^+^ has been produced after oxidation. We observed that the C-O-C stretching of TOCN-0.65 and TOCN-1.3 were mainly approximated at 1620 cm^−1^. We can further detect that the half-height width and absorption intensity of C=O feature absorption peak of the TOCN-0.65 display are relatively broader and lower than those of TOCN-1.3. Due to the overlap issue, the vibration characteristic absorption peaks at ca. 1650 cm^−1^ of COO^−^Na^+^ from TOCNs can still be distinguished as shoulders. From these results, we can infer that TOCN-1.3 has more C=O contents. In addition, the absorption region of hydroxyl groups from the pristine cellulose was shifted to about 3400 cm^−1^, showing that formation of strong hydrogen bonding between OH and C=O led to blue displacement. The degree of oxidation (DO) on cellulose C6-OH moiety can be analyzed by conductivity titration method. The measurements are shown in Figure S1 (see the Electronic Supporting Information (ESI)). We obtained DO_TOCN-0.65_ and DO_TOCN-1.3_ were 9.5% and 17.8%, respectively. These results are consistent to the FT-IR observations. Namely, a proper concentration of NaClO_(aq)_ can afford high DO and high contents of carboxylic acid sodium salt groups on the nanocelluloses.

**Figure 2:**
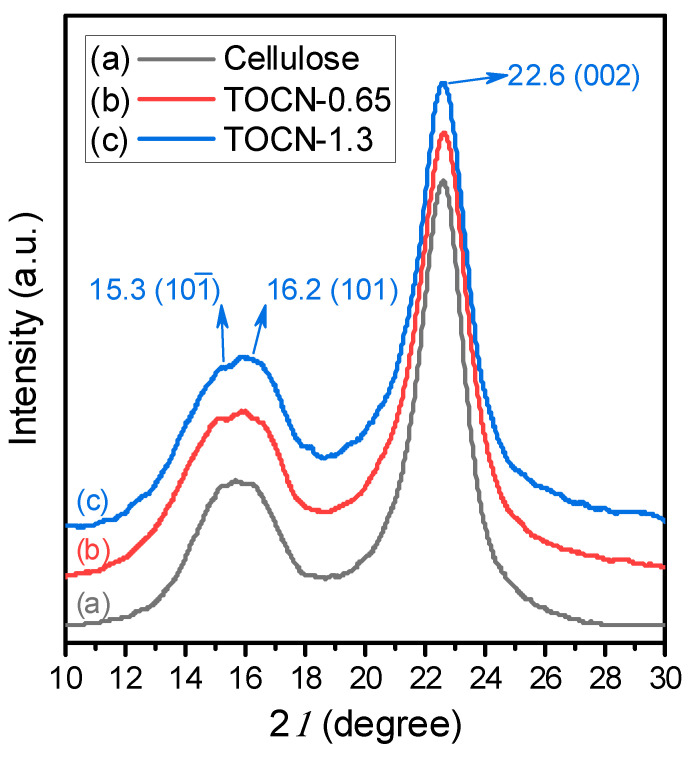
Powder XRD profiles of (a) cellulose pulp, (b) TOCN-0.65, and (c) TOCN-1.3.

Figure 2 displays powder X-ray diffraction (XRD) profiles of pristine cellulose and TOCNs. It has been reported that the cellulose II structure is comprised of 2θs of 20.6° and 22.3°. In our case, the curve a in Figure 2 (i.e., pristine cellulose) mainly demonstrated cellulose I structure, corresponding 2θs of 15.3°, 16.2°, and 22.6° to lattices of 101, 10-1, and 002. The curves b and c in Figure 2 (i.e., TOCNs) showed similar diffraction patterns. We can observe that the peaks at 15.3° and 16.2° possess a slightly larger value of full width at half maximum (FWHM_TOCNs_ = ca. 4.4°) compared to the pristine cellulose (FWHM_Cellulose_ = ca. 3.1°). Meanwhile, we also can observe that the peak at 22.6° displayed similar phenomena (i.e., FWHM_TOCNs_ = ca. 2.2° and FWHM_Cellulose_ = ca. 1.7°). It is commonly recognized that the signals from amorphous region of cellulose exist in between 18.5–20.6° [41,42]. From the results of FT-IR and XRD, we can comprehend that the oxidation reaction of cellulose causes a slight decrease in crystallinity but does not significantly affect the crystal structures. It is, thus, rational that the carboxylic acid sodium salt moieties (i.e., COO^−^Na^+^) are mainly presented on the crystal surface and in the amorphous region [43].

**Figure 3:**
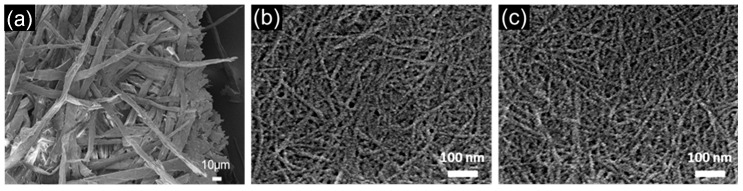
SEM images of (**a**) cellulose pulp, (**b**) TOCN-0.65, and (**c**) TOCN-1.3.

We then analyzed the morphology of cellulose before and after TEMPO oxidation method using SEM. Figure 3a represents the SEM image of pristine cellulose. We observe random distributions of the fibril directions. The width of pristine cellulose is in a range of 10–20 μm. It is plausible that these large widths of cellulose fibers would cause visible light scattering after preparations of paper sheets and lead to the formation of white opaque appearance. Figure 3b,c display the SEM images of TOCN-0.65 and TOCN-1.3 samples and the corresponding widths of TOCN-0.65 and TOCN-1.3 were significantly decreased from original micron-size to nano-size. By using different initial concentrations of NaClO_(aq)_, we observed average widths with ca. 13.9 and 11.5 nm, respectively. These results indicated that proper NaClO_(aq)_ initial concentrations would lead to obtain nano-sized fibril diameters. With the degree of oxidation approximately over 10%, sufficient amounts of surficial COO^−^Na^+^ functional groups can provide repulsive forces in between oxidized cellulose fibers. After drying the samples, we acquired anisotropic nano-fibril structures.

**Figure 4:**
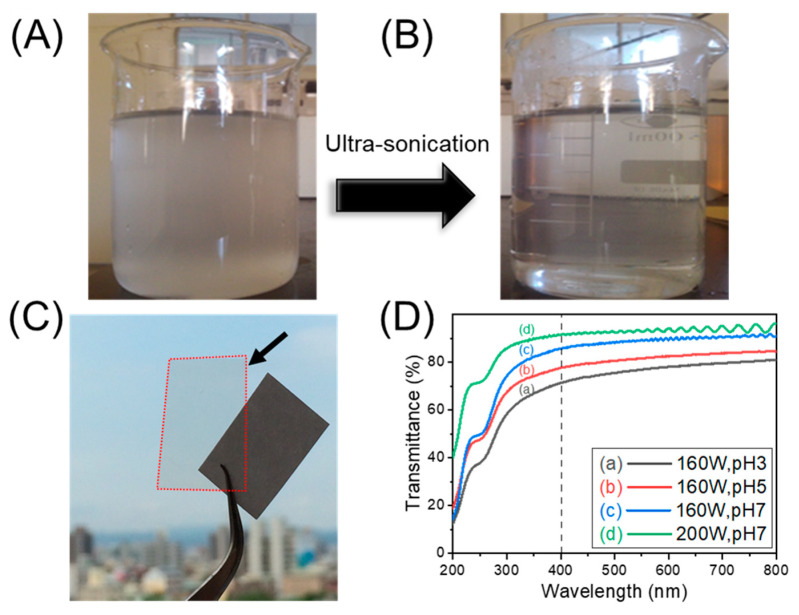
Appearances of TOCN aqueous solution (**A**) before and (**B**) atfer ultra-sonication. (**C**) Appearances of cellulose- and TOCN-made films. (**D**) UV–Vis measurements of TOCN-made films with various treated conditions.

While the obtained TOCN-1.3 was dispersed in water by regular stirring, the mixture appears with translucent turbidity (as shown in Figure 4A). With 5 min of ultra-sonication, the TOCN aqueous solution presents a transparent status (as shown in Figure 4B). These indicated that strong sonication can effectively disperse the TOCNs in water. We, thus, further studied various powers of ultra-sonication (i.e., 160 and 200 W) for 5 min and pH (i.e., 3, 5, and 7) conditions on the influences of TOCN dispersed in aqueous solution. With these various conditions, all the TOCN-made films showed similar appearances. A representative sample is demonstrated in Figure 4C. We can easily distinguish that the TOCN-made film has high transparency, but the pristine pulp-made film is opaque. We further quantitatively analyzed the transmittance (*T*) of the TOCN-made films by UV–Vis spectroscopy and the corresponding results are shown in Figure 4D. When the TOCN aqueous solution with high pH value was treated with 160 W ultra-sonication (i.e., curves a, b, and c in Figure 4D), the resulted films possessed high transmittance. In the abovementioned, we applied a mild oxidation condition with pH = 10 to prepare TOCN and rendered the surfaces of nanofibers with function groups of COOR (R = H or Na) from the C6-OH position of the glucose unit. With proper degree of oxidation of the TOCN, the higher pH values (e.g., pH = 7, curve c in Figure 4D) led to more amounts of COO^−^Na^+^ on surfaces that provide stronger repulsion forces among nanofibers. With the aid of ultra-sonication, thus, the TOCN solution with high pH value could prepare good transmittance films (i.e., *T* > 80% at λ = 400 nm). In the cases of pH = 3 and 5 (i.e., curves a and b), less amounts of COO^−^Na^+^ groups provide less repulsion forces, leading to certain influences on nanofiber aggregations. We, thus, obtained low transmittance films (i.e., *T* < 80% at λ = 400 nm). We further increased the ultra-sonication power to 200 W to treat the TOCN solution with pH = 7. As shown in curve d, we acquired a TOCN-made film with high transmittance of (i.e., *T* > 90% at λ = 400 nm). These results indicated that proper pH value and ultra-sonication power could fabricate optically transparent TOCN paper films.

**Figure 5:**
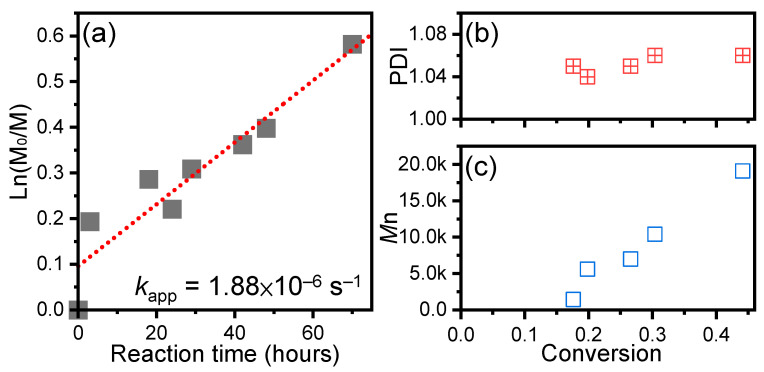
Kinetic profiles of SI ATRP from OP-Br mediated by HEBiB free initiator: (**a**) Ln vs. t and (**b**,**c**) *M*_n_, PDI vs. conversion plots (St/HEBiB/CuBr/PMDETA = 200:1:1:1; [St]_0_ = 2.6 M at 70 °C).

**Figure 6:**
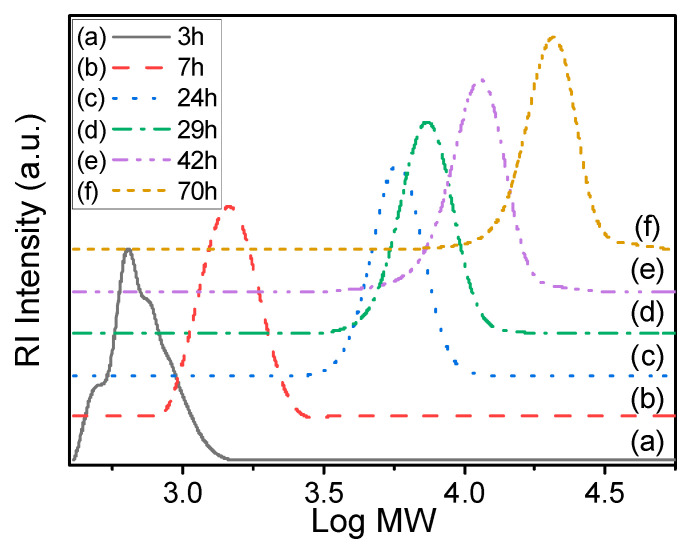
GPC traces of the corresponding SI ATRP from OP-Br film in Figure 5.

The TOCN-made optically transparent paper (OP) films possess a hydrophilic surface due to the presence of large amounts of polar groups that would restrict their applications. After oxidation reactions, however, the remaining hydroxyl groups of the D-glucose repeat units can be easily attached with hydrophobic molecules. Before our next attempt to immobilize surface initiator for surface-initiated atom transfer radical polymerization (SI ATRP), we examined the dispersibility of TOCN-1.3 in solutions to find a proper medium to conduct esterification. Figure S2 (see the ESI) shows the dispersions of TOCN-1.3 in various organic solvents. In the cases of using *N*,*N*-dimethylacetamide (DMAc), *N*,*N*-dimethylformamide (DMF), and dimethyl sulfoxide (DMSO) as solvents, we can observe good dispersion, which might be due to their high polarity and similar relatively high density to nanofibers. We can obtain quite stable dispersion solutions even after two weeks. In the cases of anisole and tetrahydrofuran (THF) as solvents, they display obvious settlement behaviors due to their relatively low polarity and density. Especially in the case of anisole, the mixture showed a distinct white turbidity, indicating that TOCN might have significant aggregations up to several hundred nanometers and cause scattering of visible light. Thus, DMAc is selected as a proper solvent for the esterification step using 4-dimethylaminopyridine (DMAP) as a catalyst and TEA as a trapping agent for acidic byproduct. In the abovementioned Scheme 1a, we immobilized an ATRP initiating site of α-bromoisobutyryl group on the OP films that could further conduct surface polymerization. As shown in Figure S3, we examined variations of the surface compositions of the cellulose-based paper films by X-ray photoelectron spectroscopy (XPS). Compared to that of the pristine cellulose-made film (i.e., curve a in Figure S3), the surface of the TOCN-made OP film (i.e., curve b in Figure S3) displays distinct binding energy of Na 1s signal at 1070 eV. After surface esterification (i.e., curve c in Figure S3), suppression of Na 1s signal and appearance of Br 3d signal at 30 eV were both distinguished, proving that parts of the OP surface OH groups were converted to an α-bromoisobutyryl group. As displayed in Scheme 1b, we further conducted SI ATRP from the obtained OP-Br film with styrene (St) to enrich their practical uses. We then used a free initiator, 2-hydroxyethyl 2-bromoisobutyrate (HEBiB), which has a similar initiating structure to monitor and control the surface polymerization facilely [44,45]. Herein, HEBiB served as a free/sacrificial initiator in the solution. It provides several important purposes: (1) HEBiB has the same analog of ATRP initiating site to the TOCN surface of isobutyryl bromide; (2) HEBiB has a similar polar property to that of the nanocellulose due to the presence of its highly polar OH group; (3) ratios of [St]_0_/[HEBiB]_0_ render control over the grafted lengths as well as the conversions due to the contents of initiating sites on the OP film should be very minor regarding the contents of the free initiator in the reaction medium; (4) the molecular weight and PDI of the grafted polymer chains can be traced from the free HEBiB initiator. Figure 5 displays kinetic plots of the SI ATRP from OP-Br film with St in toluene at 70 °C (St/HEBiB/CuBr/PMDETA = 200:1:1:1 with OP-Br (ca. 0.3 g); [St]_0_ = 2.6 M). As displayed in Figure 5a, we observed a pseudo-first order reaction trend. We acquired a slow apparent reaction rate constant (*k*_app_ = 1.88 × 10^–6^ s^−1^) due to that such moderate reaction temperature has a low propagation rate constant (*k*_p,St(70 °C)_ = ca. 500) [46]. As displayed in Figure 5b,c, all of the PDI values are below 1.07 and the molecular weight (MW) evolutions show linear growth with respect to conversions, respectively. Figure 6 shows the corresponding GPC traces during the SI ATRP from the OP-Br film with St in Figure 5. In various reaction periods, gradual increases of MWs to ca. 20k were observed. Mono-modal peaks with low distribution profiles (i.e., PDIs < 1.07) are clearly evidenced during the reactions except for the initial sample of trace a (i.e., 3 h). The shoulders in trace a might be due to the necessary stage prior to form steady-state polymerization. The influence of the content of immobilized initiating groups on the reaction period can be neglected. In addition, we did not observe any obvious termination of combination during SI ATRP. After 70 h, we obtained a conversion of ca. 0.5 with *M*_n_ = 19200 and PDI = 1.06. These results indicate a typical controlled/living radical polymerization fashion.

**Figure 7:**
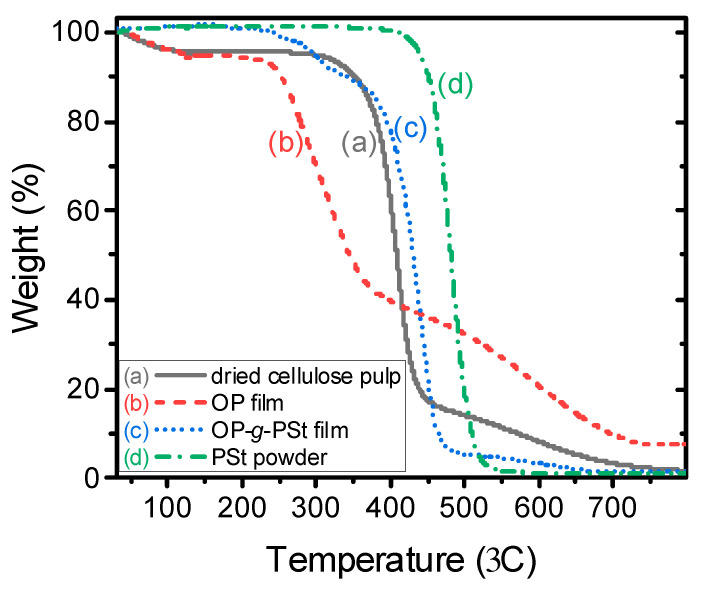
TGA profiles in a range of 25–800 °C of (a) dried cellulose pulp, (b) OP film, (c) OP-g-PSt film, and (d) PSt powder (ramp: 20 °C/min under N_2(g)_).

The thermal stability of pristine cellulose and the OP samples was exmined by thermogravimetric analysis (TGA) and shown in Figure 7. In the cases of dried cellulose pulp and OP film (i.e., curves a and b in Figure 7), we both observed obvious weight drops with 3–5 wt% loss about 110 °C. This is ascribed to that both possess plenty of OH or COO^−^Na^+^ polar groups exposed on their surfaces that could provide numerous and dense hydrogen bonding to adsorb water molecules. After release of water molecules until the temperature exceeds 225 °C, the cellulose pulp film starts significant weight loss from approximated 350 °C and has maximum weight loss decomposition rate (−*r*_dec(max)_ = 1.16 wt%/°C) at about 400 °C. However, the OP film starts significant weight loss from approximately 270 °C and follows by having −*r*_dec(max)_ = 0.46 wt%/°C at about 300 °C. In comparisons, the OP film has less thermal stability mainly due to its possessions of less thermal stable COO^−^Na^+^ polar groups on the surfaces. After SI ATRP of St from the OP film, the obtained OP-*g*-PSt film (i.e., curve c in Figure 7) performs a two-stage degradation profile. The minor first stage starts weight loss from approximately 270 °C and has −*r*_dec(max)_ = 0.18 wt%/°C at about 290 °C. The major second stage starts significant weight loss from approximately 390 °C and has −*r*_dec(max)_ = 1.08 wt%/°C at about 420 °C. The first degradation stage is presumably contributed from the less stable part of cellulose. The second degradation stage should be contributed from the grafted PSt bush layer on the OP film. In the case of PSt homopolymer (i.e., curve d in Figure 7), we observe single step degradation that starts significant weight loss from approximately 430 °C and follows by having −*r*_dec(max)_ = 1.64 wt%/°C at about 490 °C. With grafted PSt chains on OP film surface, namely, the nanocomposite significantly improved the thermal stability. Besides, we observe the disappearance of water releasing at about 110 °C that is due to the coverages of hydrophobic PSt chains and result in less water adsorption property. Compared with these profiles, thus, we successfully attained OP-*g*-PSt film.

**Figure 8:**
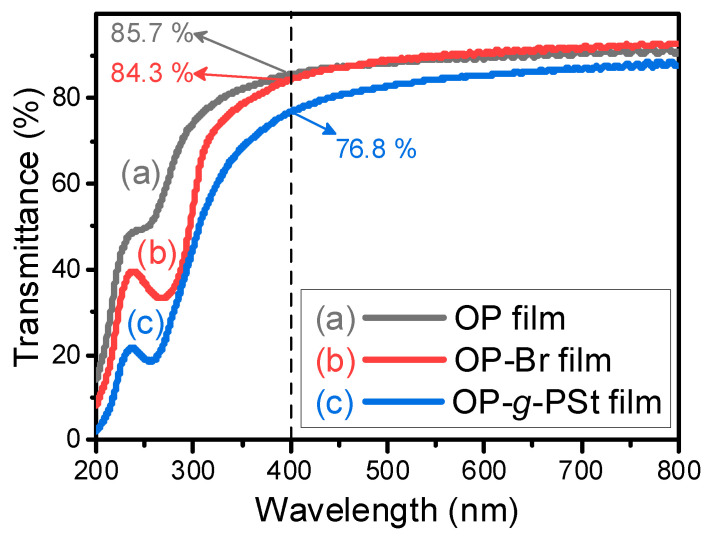
UV–Vis measurements (λ = 200–800 nm) of (a) OP, (b) OP-Br, and (c) OP-g-PSt films.

After successfully rendering various functional groups/chains on the surfaces, we then analyzed the UV–Vis adsorption spectra of the (modified) OP films and Figure 8 displays the results. Compared to the OP and OP-Br films (i.e., curves a and b in Figure 8), we acquired both high transmittance (*T*_@400nm_ > 84%) and a wavelength larger than 400 nm. However, we observe a significant decrease of the OP-Br film that might be due to the influences from the surface attached α-bromoisobutyryl groups. In the case of OP-*g*-PSt sample (i.e., curve c in Figure 8), we acquired a moderate decrease of transmittance at 400 nm (*T*_@400nm_ = ca. 77%) compared to the first two samples. In the region of large wavelength, oscillations were notably observed. The oscillating signals presumably resulted from the slight wrinkle of the OP surface scattering. In the region of low wavelength (i.e., <400 nm), we observe a significant transmittance decrease that is ascribed to the strong adsorption nature of the surface PSt chains.

**Figure 9:**
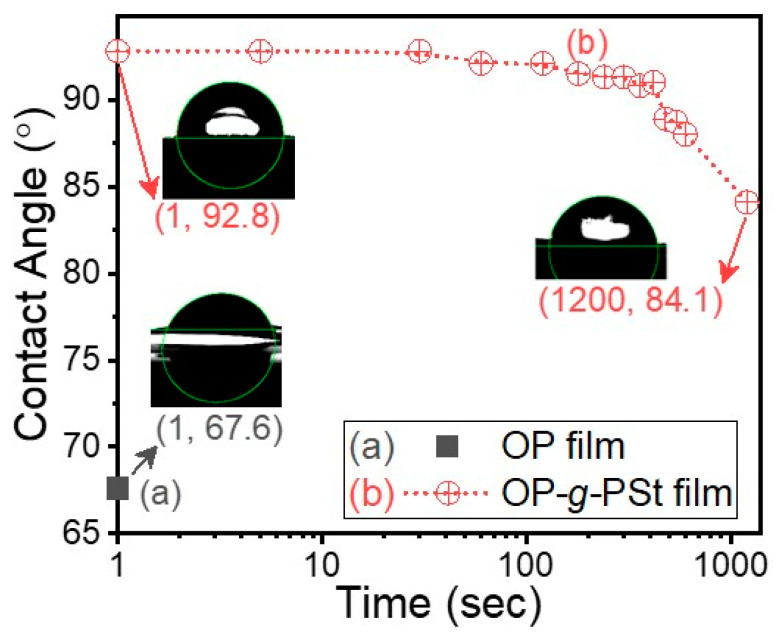
WCA measurements and images of (a) OP and (b) OP-g-PSt films.

We then measured the hydrophobicity of the OP and OP-*g*-PSt film surfaces by water contact angle (WCA) analysis. In the case of OP film (i.e., datum a in Figure 9), the WCA was initially revealed from the image with 67.6°. Then the water droplet quickly penetrated the film by the capillary forces within cellulose fibers and disappeared after a few seconds, indicating its hydrophilic surface. In the case of OP-*g*-PSt film (i.e., trend b in Figure 9), the initial WCA image is at 92.8°. Although the WCAs gradually decreased, the sample still possessed a WCA value of about 85° after 15 min. There might be two reasons. One is that the PSt coverage of part of the surface graft is imperfect and a small amount of the water droplet could slowly penetrate the inner layer or be pulled into the film by capillary force. Another reason is that while the water droplet gradually volatilized, the overall volume of the droplet slightly decreased. During the vaporization, the original periphery of the water droplet would have residual interfacial attraction force resulting in the deformation of the droplet. Then we acquired a slight decrease of the WCAs. In short, the grafted PSt chains significantly render the surface with hydrophobicity.

## 4. Conclusions

In this study, we successfully oxidized pristine celluloses with the TEMPO oxidation method to obtain TOCN with high crystallinity characterized by FT-IR, SEM, and XRD. We can further control/increase the degree of oxidations of the cellulose fibers (i.e., from 9.5% to 17.8%) using different concentrations of NaClO_(aq)_. Having the nano-sized cellulose fibers (ca. 11.5 nm), we, thus, prepared high transmittance and optically transparent paper (OP) films and subsequently modified the surface with ATRP initiating sites of the α-bromoisobutyryl group, which are confirmed by UV–Vis and XPS measurements, respectively. We subsequently conducted SI ATRP with St to afford OP-*g*-PSt film. In the presence of a free initiator, we can attain a typical controlled/living radical polymerization fashion. The obtained OP-*g*-PSt performed property enhancements in thermal stability and hydrophobicity compared to that of pristine OP film. Our study provides a novel approach of the preparations of large-sized TOCN optical films, their effective surface modifications, good control of grafting polymer chains by SI ATRP, and alternations of the physical and surface properties.

## Data Availability

The data presented in this study are available on request from the corresponding author.

## Supplementary Materials

The following are available online at https://www.mdpi.com/article/10.3390/polym14050946/s1, Figure S1: Measurements of degree of oxidation (DO) of (a) TOCN-0.65 and (b) TOCN-1.3 samples via titration methods. Figure S2: Dispersion examinations of TOCN-1.3 in various polar organic solvents. Figure S3: XPS patterns of (a) cellulose-made, (b) TOCN-made OP, and (c) OP-Br films.
