# Supplementary material for: SI ATRP for the Surface Modifications of Optically Transparent Paper Films Made by TEMPO-Oxidized Cellulose Nanofibers
Source: Polymers (Basel). 2022 Feb 26;14(5):946. doi: 10.3390/polym14050946 (PMC8912791; doi:10.3390/polym14050946)
Supplement: Supplementary file 1 [file polymers-14-00946-s001.zip › polymers-1606380-supplementary.pdf]

polymers

MDPI

Electronic Supplement Information (ESI) for

SI ATRP for the Surface Modifications of Optically Transparent Paper Films Made by TEMPO-Oxidized Cellulose Nanofibers

Jem-Kun Chen †1, Hsiang-Ya Huang †2, Cheng-Wei Tu [3], Li-Ting Lee [4], Tongsai Jamnongkan [5,*], and Chih-Feng Huang [2,*]

.

1 Department of Materials Science and Engineering, National Taiwan University of Science and Technology, Taipei 10607, Taiwan; jkchen@mail.ntust.edu.tw (J.-K.C.)
2 Department of Chemical Engineering, i-Center for Advanced Science and Technology (iCAST), National Chung Hsing University, Taichung 40227, Taiwan; sylvia19971118@gmail.com (H.-Y.H.)
3 Industrial Technology Research Institute, Chutung, Hsinchu 31057, Taiwan; CWTu@itri.org.tw (C.-W.T.)
4 Department of Materials Science and Engineering, Feng Chia University, Taichung 40724, Taiwan; ltlee@fcu.edu.tw (L.-T.L.)
5 Department of Fundamental Science and Physical Education, Faculty of Science at Sriracha, Kasetsart University, Chonburi 20230, Thailand

* Correspondence: jamnongkan.t@ku.ac.th (T.J.); HuangCF@dragon.nchu.edu.tw (C.-F.H.)

Captions:

Fig. S1. Measurements of degree of oxidation (DO) of (**a**) TOCN-0.65 and (**b**) TOCN-1.3 samples via titration methods.

Fig. S2. Dispersion examinations of TOCN-1.3 in various polar organic solvents.

Fig. S3. XPS patterns of (**a**) cellulose-made, (**b**) TOCN-made OP, and (c) OP-Br films.

Experimental

1. Materials.

St (99%) was purchased from Acros and purified by passing through a column filled with basic alumina to remove the inhibitor. Wood pulp (LBKP grade) was provided by Chung Hua Pulp. 2,2,6,6-Tetramethylpiperidine 1-oxyl (TEMPO, 98%+), 4,4-dimethylaminopyridine (DMAP, 99%), 2-bromoisobutyryl bromide (BiB, 97%), and copper(I) bromide (CuBr, 98%) were purchased from Sigma–Aldrich (St. Louis, MI, USA). *N,N,N´,N´´,N´´*-Pentamethyldiethylenetriamine (PMDETA, 99%), and triethylamine (TEA, 99.5%) were purchased from Alfa Aesar. NaOH (98%), KBr (99%), HCl (35%), NaBr (99.5%), and $NaClO_{(aq)}$ (12%) were purchased from Showa (Saitama, Japan). CuBr was purified by washing with glacial acetic acid, filtered, washed with anhydrous ethanol, and dried under vacuum. All solvents were distilled before use.

2. Preparations of TEMPO-oxidized cellulose nanofiber (TOCN).

TEMPO (1.0 g, 6.4 mmol) and NaBr (8.0 g, 78.2 mmol) were added to wood pulp (8.0 g) dispersed in deionized water (DIW, 600 mL). 1 M $NaOH_{(aq)}$ was added slowly until the mixture achieved pH = 10, and then proper amounts of $NaClO_{(aq)}$ was added to begin the oxidation-reduction reactions. After a period of time, the reaction was stopped by diluting with DIW. The product was purified by repeating the DIW washing/high-speed-centrifugation for a few cycles, till the solution turned to about pH = 7. The solution was concentrated and a gel-type TOCN sample was acquired (4.9 wt% of TOCN solid content; yield: 92.4%).

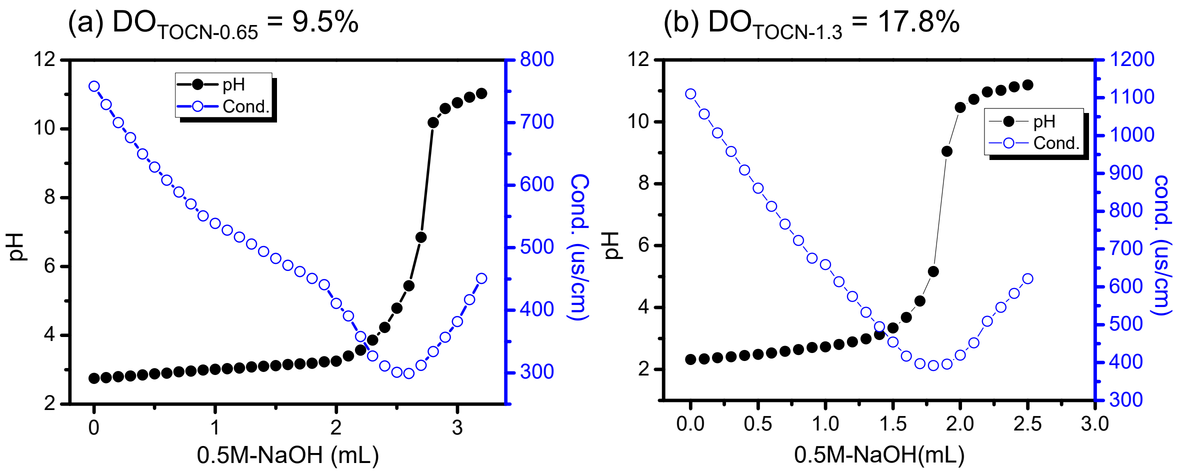

Figure S1. Measurements of degree of oxidation (DO) of (**a**) TOCN-0.65 and (**b**) TOCN-1.3 samples via titration methods.

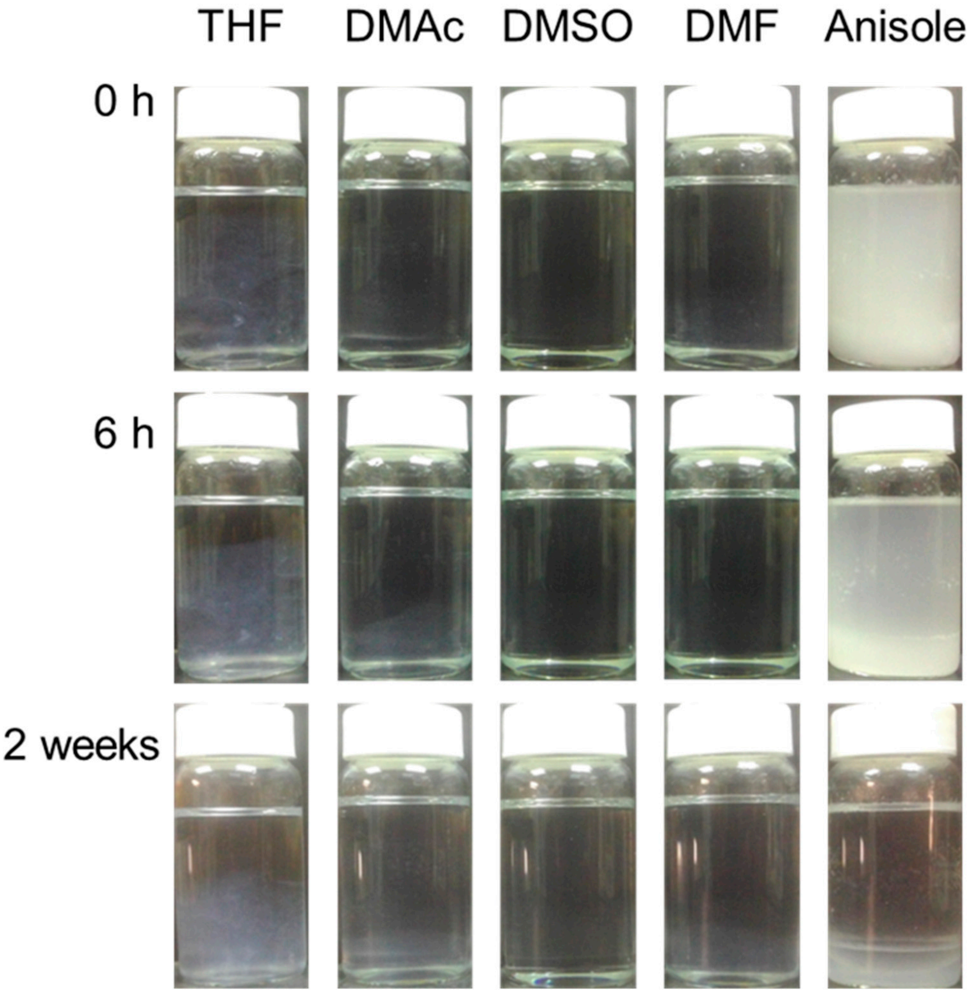

Figure S2. Dispersion examinations of TOCN-1.3 in various polar organic solvents.

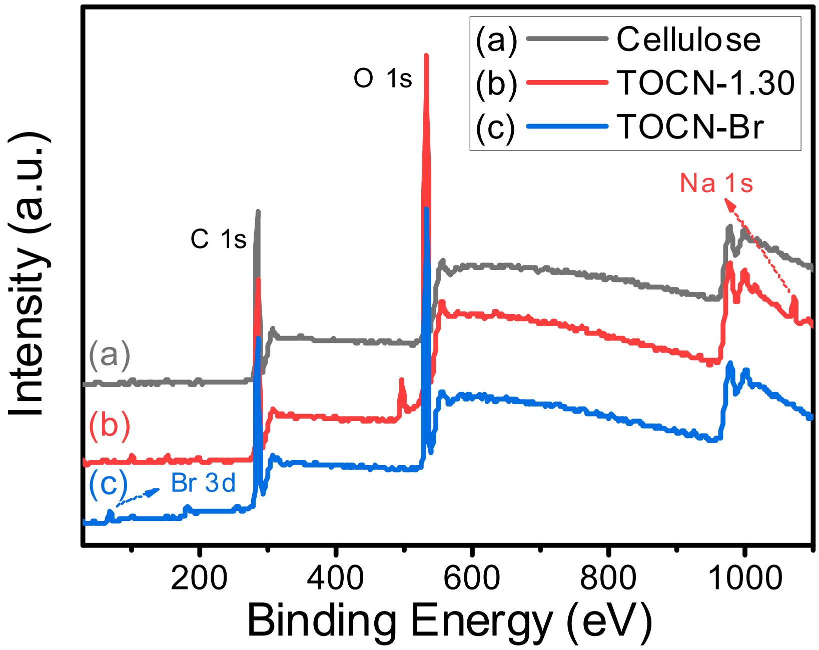

Figure S3. XPS patterns of (**a**) cellulose-made, (**b**) TOCN-made OP, and (**c**) OP-Br films.
